# Chondroblastoma Affecting the Apophysis of the Greater Trochanter in a Child

**DOI:** 10.7759/cureus.34908

**Published:** 2023-02-13

**Authors:** Svetoslav A Slavchev, Philip J O'Connor, Georgi P Georgiev

**Affiliations:** 1 Orthopaedics and Traumatology, Medical University of Sofia, Sofia, BGR

**Keywords:** pediatric orthopedic surgery, denosumab, growth plate, physis, bone grafting, synthetic bone substitute, greater trochanter, apophysis, chondroblastoma

## Abstract

Chondroblastomas are rare primary bone tumours typically affecting the epiphyses and less frequently the apophyses of the growing skeleton. Most cases are treated by intralesional curettage with or without local adjuvants and this technique can produce good long-term outcomes. Herein, we describe a case of chondroblastoma of the greater trochanter in a 12-year-old male child that was treated by intralesional curettage and grafting with calcium phosphate bone cement (Neocement Inject® P, Bioceramed, Loures, Portugal). A brief review of the literature is also presented.

## Introduction

Chondroblastomas are rare cartilaginous tumours arising typically in the epiphyses or apophyses of long bones, usually the proximal humerus and around the knee [1]. Currently, it is defined by the World Health Organisation as “a benign tumour of bone that has a predilection for epiphyseal or apophyseal regions, composed of chondroblastic cells and islands of eosinophilic chondroid matrix” [2]. In spite of its benign histological nature, metastases to the lungs, bones, and soft tissues have been reported [3]. Open surgery is the principal treatment modality but curative percutaneous radiofrequency thermoablation or cryoablation has also been reported [4,5]. Rarely, in multiple recurrences or malignant transformation, amputation could be considered [3]. The reported recurrence rates after curettage range from 9.5% to 32% [6].

## Case presentation

A 12-year-old Caucasian male child was brought to our institution complaining of pain and limited motion in his left hip. The symptoms had a duration of about a year. The patient reported that initially the pain was mild and occurred only during physical activities, followed by worsening limitation of motion in the hip joint. Over the course of several months, it had become severe and constant, virtually insusceptible to non-steroid anti-inflammatory medication.

Clinical examination revealed the inability of weight-bearing of the affected limb, hypotrophy of the gluteal and thigh muscles, and severe pain in the trochanteric region, which was extremely tender with a normal appearance of the overlying skin.

**Figure 1:**
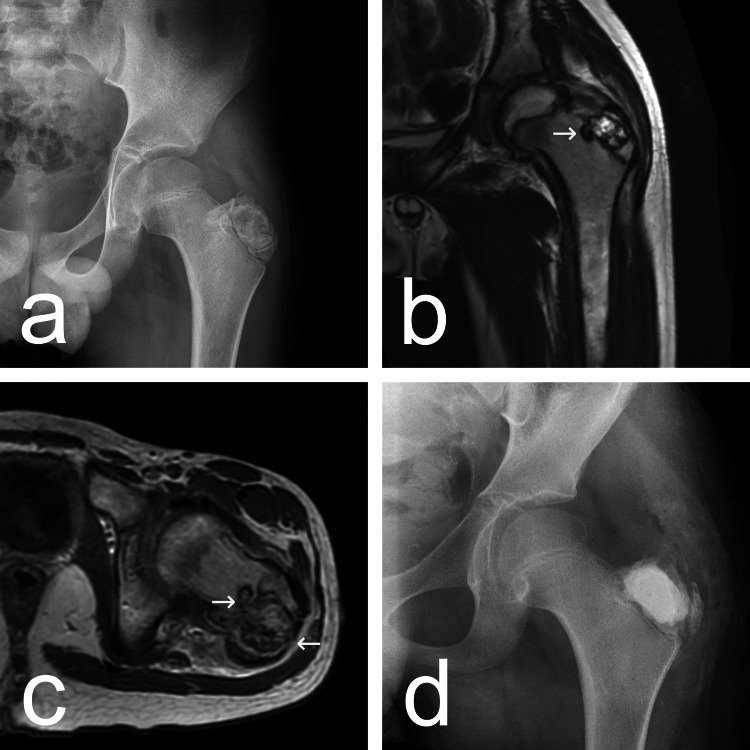
Imaging studies before and after surgery. 1a: Plain radiograph (anteroposterior) of the proximal femur at presentation. 1b, 1c: Frontal oblique and axial T1 weighted magnetic resonance tomography (MRT) images of the proximal femur at presentation. The growth plate and the lateral cortex are breached (arrows). 1d: Plain radiograph (anteroposterior) of the proximal femur after surgery.

On plain radiography, the greater trochanter appeared to be somewhat enlarged as compared to the normal side and almost completely occupied by an ovoid lesion with radiodense and radiolucent areas surrounded by an osteosclerotic rim (Figure 1a). On magnetic resonance tomography (MRT), the lesion was well-demarcated, heterogeneous, slightly lobulated, and breaching both the growth plate and the lateral cortex of the greater trochanter (Figures 1b, 1c).

**Figure 2:**
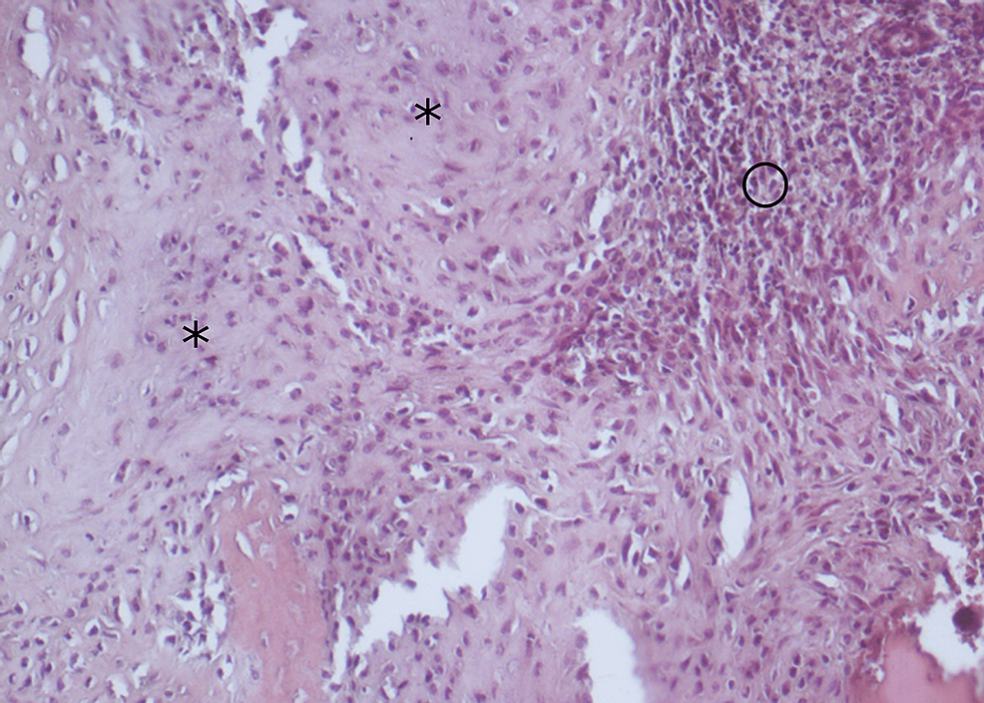
Hematoxylin and eosin staining of chondroblastoma (×200). Lobules of chondroid matrix (asterisks) populated by irregularly shaped chondroblasts adjacent to hypercellular areas (circle) with histiocyte-like cells. Multinucleated giant cells that are typically a feature of chondroblastomas are not present in this slide.

As it was deemed that the lesion showed signs of local aggressiveness, an open biopsy was performed through a lateral approach and the diagnosis of chondroblastoma was made (Figure 2).

In the second stage, the lesion was thoroughly curetted and rinsed with hydrogen peroxide as an adjuvant. The bone defect was filled with calcium phosphate bone cement (Neocement Inject® P, Bioceramed, Loures, Portugal) (Figure 1d) and the wound was closed in the usual manner. The postoperative course was uneventful with a resolution of pain and gradual restoration of limb function. The patient was lost to follow-up after one month.

## Discussion

Chondroblastoma is a rare benign bone tumour that represents less than 1% of all primary bone tumours occurring mainly in the epiphyses, and less frequently in the apophyses, of the immature skeleton with 60% of cases developing in the second decade of life and with a male predilection of 2-3:1 [7]. Their purely metaphyseal location is exceptionally rare [7]. They could, however, develop in any skeletal location [8].

Chondroblastomas are usually symptomatic and rarely discovered incidentally on plain radiographs [1]. The typical symptoms are pain and local tenderness followed by swelling and limitation of motion in adjacent joints [9].

While the clinical and imaging features might suffice for making a precise diagnosis in most cases, the differential diagnosis includes other tumours such as giant cell tumour of bone (GCTB) and aneurysmal bone cyst (ABC) that might share some common characteristics in clinical presentation, and radiological and even histological appearance, especially multinucleated giant cells and hemosiderin deposits [1]. An H3K36M mutation in either *H3F3A* or *H3F3B* gene has been discovered which is 70-95% specific for chondroblastoma [1]. In GCTB, another mutation H3G34W in *H3F3A* gene has been discovered that can be detected through immunohistochemistry and is deemed to be specific to this tumour [10]. In primary ABCs, typical translocations t(16; 17) (q22; p13) and 7(17; 17) (q22; p 13) have been identified while in secondary ABCs that are present alongside chondroblastomas or other tumours, no genetic abnormalities exist [11].

Surgery is the principal treatment modality, and aggressive curettage has been advocated despite the proximity of the growth plate, followed by packing the defect with bone graft, synthetic bone substitute, or polymethylmethacrylate bone cement [1,9]. Compared to morselized bone graft or calcium phosphate granules, calcium phosphate bone cement has the advantage of superior mechanical strength and, furthermore, its homogeneity allows for easier radiographic detection of a local recurrence. Radiofrequency ablation has also been used as a primary treatment of chondroblastoma but the mechanical failure of weight-bearing articular surfaces is possible as well as thermal damage of uninvolved compartments of the joint [12]. Cryosurgery, too, has been used, both as an adjuvant to intralesional curettage (liquid nitrogen) and as a standalone procedure (percutaneous cryoablation), in the treatment of chondroblastoma [5,13].

Local recurrence rates after treatment vary considerably and the presence of an ABC component, biologic aggressiveness, atypical location, and the presence of an open physis are thought to increase the risk of recurrence [6]. According to Zekry et al., the risk factors include tumour location around the hip joint, an active physis, incomplete removal, and aggressive behaviour of the lesions [8]. However, some authors argue that an open physis is not a risk factor for recurrence per se but rather its presence might discourage the surgeon to perform sufficiently aggressive curettage [9].

As local recurrence is considered to be a major risk factor for metastasising, since metastatic disease virtually always develops after at least one episode of local recurrence, it is advisable that patients with local recurrences undergo a total work-up similar to that for a malignant disease [3,14]. Recently, the use of denosumab has been reported in cases of metastatic chondroblastoma to the lungs [14,15].

Due to the epiphyseal location of most chondroblastomas, late post-surgical complications may occur such as limb length discrepancies, angular deformities, and osteoarthritis [8]. The management of these complications may require additional surgery, e.g. limb lengthening, correction osteotomies, or arthroplasty [16]. The interposition of polymethylmethacrylate bone cement across the growth plate has been proposed as a means of preventing premature physeal closure [6].

## Conclusions

Herein, we described an interesting case of a chondroblastoma that affected the apophysis of the greater trochanter in a child. The treatment included extended intralesional curettage and grafting with calcium phosphate bone cement. Using such a graft substance is an excellent option because of early restoration of the mechanical properties of the bone together with good potential for bone remodelling and earlier detection of an eventual local recurrence.

Despite the benign histological characteristics and usually uncomplicated clinical course of chondroblastoma, its biological aggressiveness should not be underestimated by clinicians, especially in cases of a local recurrence.
